# Plasma concentration of dexmedetomidine combined with fixed remifentanil for nociceptive and hemodynamic control during skull pin fixation

**DOI:** 10.1038/s41598-026-50692-y

**Published:** 2026-04-27

**Authors:** Wei-Cheng Tseng, Ann‑Shung Lieu, Yu-Chi Tu, Yu-Feng Su, Tsai-Shan Wu, Yi-Wei Kuo, Zhi-Fu Wu

**Affiliations:** 1 Department of Anesthesiology, Tri-Service General Hospital, National Defense Medical University, Taipei, 114 Taiwan; 2 Department of Surgery, Division of Neurosurgery, Kaohsiung Medical University Hospital, Kaohsiung Medical University, Kaohsiung, 807 Taiwan; 3 Department of Surgery, Faculty of Medicine, College of Medicine, Kaohsiung Medical University, Kaohsiung, 807 Taiwan; 4 Department of Anesthesiology, Kaohsiung Medical University Hospital, Kaohsiung Medical University, No. 100, Tzyou 1st Road, Sanmin District, Kaohsiung, 807 Taiwan; 5 Department of Anesthesiology, Faculty of Medicine, College of Medicine, Kaohsiung Medical University, Kaohsiung, 807 Taiwan; 6 Center for Regional Anesthesia and Pain Medicine, Wan Fang Hospital, Taipei Medical University, Taipei, 116 Taiwan

**Keywords:** Analgesia nociception index, Dexmedetomidine, Remifentanil, Skull pin fixation, Target-controlled infusion, Total intravenous anesthesia, Medical research, Neurology, Neuroscience

## Abstract

Skull pin fixation during neurosurgery induces intense nociceptive and hemodynamic responses. Dexmedetomidine is commonly used in anesthesia to enhance perioperative stability; however, its optimal plasma concentration (Cp) when combined with remifentanil at a fixed effect-site concentration (Ce) remains undefined. This prospective study enrolled 24 patients undergoing intracranial surgery to determine the 95% effective concentration (EC_95_) of dexmedetomidine for skull pin fixation using the analgesia nociception index (ANI) and hemodynamic parameters. Anesthesia was maintained with propofol and remifentanil (Ce: 2.0 ng/mL), with dexmedetomidine administered via target-controlled infusion (TCI). The initial Cp of dexmedetomidine was set at 0.4 ng/mL and adjusted in 0.05 ng/mL increments using a modified up-and-down method. Analgesia was deemed successful if ANI ≥ 30 with mean arterial pressure (MAP) and heart rate (HR) within ± 20% of baseline values. Probit regression analysis revealed EC_50_ and EC_95_ of dexmedetomidine at 0.325 ng/mL and 0.395 ng/mL, respectively, for maintaining nociceptive and hemodynamic stability. Dexmedetomidine Cp below 0.3 ng/mL were associated with greater fluctuations in ANI, MAP, and HR. ANI < 30 demonstrated a sensitivity of 81.8% and a specificity of 100% for detecting analgesic failure. These findings suggest that when remifentanil is maintained at a Ce of 2.0 ng/mL, a dexmedetomidine Cp of 0.395 ng/mL provides optimal nociceptive and hemodynamic control during skull pin fixation, supporting the use of individualized dexmedetomidine titration and ANI-guided multimodal monitoring in neurosurgical anesthesia.

**Supplementary Information:**

The online version contains supplementary material available at 10.1038/s41598-026-50692-y.

## Introduction

Skull pin fixation is a necessary but intensely noxious procedure used to immobilize the head during neurosurgical interventions. This strong nociceptive stimulus triggers profound sympathetic activation, often resulting in significant hemodynamic disturbances, including hypertension and tachycardia, if not properly managed. Acute increases in mean arterial pressure (MAP) and heart rate (HR) can be particularly harmful in neurosurgical patients with elevated intracranial pressure (ICP), as they may exacerbate ICP, precipitate brain herniation, or lead to complications such as pulmonary edema and aneurysmal rupture^1^. Given that preventing acute hypertensive responses to noxious stimuli during skull pin fixation is a critical objective in neurosurgical anesthesia, various strategies have been proposed to attenuate the hemodynamic fluctuations, including the administration of opioid analgesics and the use of local infiltration or scalp nerve blocks^2–7^. However, opioids alone are often insufficient to fully suppress these hemodynamic responses^2,3,7^. Moreover, excessive dosing can result in hemodynamic instability, which may adversely affect neurosurgical outcomes. Therefore, adjunctive agents such as dexmedetomidine have been investigated for their potential to enhance hemodynamic stability and blunt nociceptive activation during skull pin fixation.

Dexmedetomidine, a highly selective α_2_-adrenergic agonist, provides several advantages during neurosurgical procedures^8^. Its sedative and analgesic properties, along with minimal respiratory depression, contribute to hemodynamic stability and facilitate smooth emergence from anesthesia—an essential concern in neurosurgical cases requiring early neurological evaluation. Additionally, its opioid-sparing effect may reduce the incidence of postoperative nausea, vomiting, and respiratory complications. Previous studies have shown that dexmedetomidine can attenuate the hemodynamic perturbations associated with skull pin fixation^9–12^. However, dexmedetomidine is associated with dose-dependent adverse effects, particularly hypotension and bradycardia, which may impair cerebral perfusion and increase perioperative risk^13^. Rapid infusion may also cause transient hypertension due to initial peripheral vasoconstriction. Notably, pharmacodynamic variability, coupled with inconsistent timing between bolus administration and skull pin application, has resulted in uncertain and potentially fluctuating drug concentrations at the time of nociceptive stimulation. In previous studies^9,10,12^, dexmedetomidine was typically administered as a bolus without plasma concentration (Cp) targeting. A standard loading dose of 1.0 mcg/kg infused over 10 min usually produces clinical effects within 5–10 min, with peak effects occurring approximately 15–30 min after infusion. This delayed and variable peak effect makes it challenging to coordinate with the timing of skull pin insertion. Thus, the actual dexmedetomidine concentration during nociceptive stimulation becomes unpredictable, limiting reproducibility and complicating interpretation of its hemodynamic effects.

Remifentanil is an ultra-short-acting phenylpiperidine opioid with potent and selective mu-opioid receptor agonist activity. It is characterized by high lipid solubility and rapid metabolism by nonspecific plasma and tissue esterases, which facilitates a rapid onset and offset of analgesic effect^14^. Owing to its rapid onset, ease of titration, and favorable recovery profile, remifentanil is considered an ideal opioid for continuous infusion to manage pain related to surgical stimulation. When used successfully in combination with dexmedetomidine in various surgical settings^15–17^, remifentanil appears to offer baseline analgesia that prevents dexmedetomidine overdose and bridges the delay in reaching peak effect of dexmedetomidine. However, to the best of our knowledge, no previous study has investigated the optimal Cp of dexmedetomidine combined with a fixed effect-site concentration (Ce) of remifentanil for skull pin fixation under total intravenous anesthesia (TIVA) using target-controlled infusion (TCI) pumps. Recently, the analgesia nociception index (ANI) has emerged as an objective tool for monitoring intraoperative nociceptive balance and guiding analgesic titration^18–20^, demonstrating superior sensitivity compared to traditional hemodynamic indicators. Therefore, this study aimed to determine the 50% and 95% effective concentrations (EC_50_ and EC_95_) of dexmedetomidine required to achieve adequate analgesia during skull pin fixation, guided by the ANI and hemodynamic monitoring.

## Methods

### Study design and patient selection

This prospective, single-center study was conducted at Kaohsiung Medical University Hospital (KMUH), Kaohsiung, Taiwan. The study was approved by the Institutional Review Board of KMUH (KMUHIRB-F(I)-20250016) on January 10, 2025, and all methods were performed in accordance with relevant guidelines and regulations. Written informed consent was obtained from all participants prior to enrollment. The study was also registered at ClinicalTrials.gov (NCT06837818) on February 20, 2025, and conducted between March 7 and April 28, 2025.

Twenty-four adult patients scheduled for elective intracranial surgery requiring skull pin fixation under TIVA using a TCI system (Orchestra® Base Primea; Fresenius Kabi AG, Bad Homburg, Germany) with tracheal intubation were enrolled in this study. Eligible participants were between 20 and 80 years of age and had an American Society of Anesthesiologists (ASA) physical status of I to III. Exclusion criteria included: ASA physical status IV or higher; the presence of major comorbidities requiring beta-adrenergic blocker therapy; pacemaker implantation or significant arrhythmias (e.g., atrial fibrillation or bradyarrhythmias); chronic pain conditions; emergent surgery; pregnancy; and known allergy to propofol, remifentanil, or dexmedetomidine.

## Anesthesia protocol

All patients fasted overnight prior to the procedure, and no premedication was administered before the induction of general anesthesia (GA). Standard monitoring was applied to all patients, including electrocardiography (lead II), noninvasive blood pressure, pulse oximetry, end-tidal carbon dioxide (EtCO_2_), and direct radial arterial blood pressure (ABP) monitoring. In addition, all patients were monitored using the bispectral index (BIS; BIS™ Complete 2-Channel Monitor, Covidien, Boulder, CO, USA) and the ANI (PhysioDoloris™, MDoloris Medical Systems, Loos, France). Before anesthetic induction, patients were preoxygenated with 100% oxygen at 6 L/min via a facemask until peripheral oxygen saturation reached 99–100%.

GA was induced using two separate TCI pumps to deliver remifentanil at a Ce of 2.0–4.0 ng/mL (50 mcg/mL, Minto model) and propofol at a Ce of 3.0–6.0 mcg/mL (10 mg/mL, Schnider model). Rocuronium (0.6 mg/kg) was administered after loss of consciousness to facilitate tracheal intubation in all patients. Following induction, dexmedetomidine was also infused using a TCI pump (Medcaptain HP-30 Syringe Infusion Pump, Shenzhen, Guangdong, China) at a Cp of 0.2 ng/mL, based on the Dyck model^21^. During anesthetic maintenance, the intraoperative infusions of propofol and remifentanil were titrated to maintain a BIS value between 40 and 60, and an ANI, including both the instantaneous ANI (ANIi; 64-second moving average) and the mean ANI (ANIm; 4-minute moving average), between 50 and 70 throughout the procedure. An oxygen flow rate of 0.3 L/min and air at 0.7 L/min were administered. EtCO_2_ was controlled within 35–40 mmHg by adjusting the ventilation rate, with peak airway pressure kept below 30 cmH_2_O. For patients who developed hypertension (ABP > 180/100 mmHg) or tachycardia (HR > 100 beats per minute [bpm]) during GA, antihypertensive agents or beta-blockers were administered. In contrast, ephedrine or atropine was given in cases of hypotension (ABP < 90/50 mmHg) or bradycardia (HR < 50 bpm). After surgery, all patients were transferred to the intensive care unit for postoperative observation and care.

### Up-and-down sequential allocation method

The Cp of dexmedetomidine for each patient was determined using a modified Dixon’s up-and-down sequential allocation method. The initial Cp for skull pin fixation was set at 0.4 ng/mL. It was adjusted from the baseline level (0.2 ng/mL) to the target level (0.4 ng/mL) over approximately 13.6 min. Remifentanil was maintained at a Ce of 2.0 ng/mL prior to skull pin fixation. Once the targeted dexmedetomidine Cp was achieved, skull pin fixation was performed. Following fixation, the dexmedetomidine Cp was reduced back to 0.2 ng/mL, and the remifentanil Ce was decreased to 0.5 ng/mL. A step size of 0.05 ng/mL was used for dexmedetomidine titration. If the previous patient exhibited successful analgesia, the Cp for the next patient was decreased by one step; if the response was a failure, it was increased by one step. All anesthetic procedures were performed by a single anesthesiologist, while another investigator assessed each patient’s response to skull pin fixation (success or failure). Both the operating neurosurgeon and the patient were blinded to the Ce of remifentanil and propofol and the Cp of dexmedetomidine at the time of skull pin fixation.

Based on prior studies^10,12,22^, the peak Cp of dexmedetomidine was estimated using the Dyck model to fall between 0.15 and 0.6 ng/mL, a range shown to provide adequate analgesia even without opioid co-administration during skull pin fixation. In addition, remifentanil exhibits an anesthetic-sparing effect when combined with other anesthetic agents. Accordingly, we selected a conservative initial target Cp of 0.4 ng/mL for dexmedetomidine via a TCI system, in combination with a fixed remifentanil Ce of 2.0 ng/mL. This regimen aimed to ensure both hemodynamic stability and effective nociceptive control. The titration step size for dexmedetomidine was set at 0.05 ng/mL, based on the outcome (success or failure) in the preceding patient. Failure was defined as the presence of any of the following during skull pin fixation: (1) inadequate antinociception, indicated by an ANI < 30^19^; or (2) hemodynamic instability, defined as a > 20% increase in MAP or HR from baseline, ABP > 180/100 mmHg, or HR > 100 bpm^19,23^. In the absence of these conditions, the response was considered successful (Table 1).

**Table 1:** Criteria for success and failure during skull pin fixation.

Parameter	Success Criteria	Failure Criteria
ANI	ANIm and ANIi both ≥ 30 during and after fixation	ANIm or ANIi < 30 during or within 2 min after fixation
MAP	No increase > 20% from baseline	Increase > 20% from baseline or ABP > 180/100 mmHg during or within 2 min after fixation
HR	No increase > 20% from baseline	Increase > 20% from baseline or > 100 bpm during or within 2 min after fixation

*ABP* arterial blood pressure,* ANIi(m)* instantaneous (mean) analgesia nociception index,* bpm* beats per minute,* HR* heart rate,* MAP* mean arterial pressure.

### Variables

Patient demographics (age, sex, height, and weight) and clinical data (tumor location, anesthesia time, and operation time) were collected from medical records. In addition, the Ce of remifentanil and propofol, the Cp of dexmedetomidine, MAP, HR, BIS, and ANI values were recorded at five predefined time points: T_0_, baseline (at induction of GA before loss of consciousness); T_1_, 2 min before skull pin fixation; T_2_, during skull pin fixation; T_3_, 5 min after skull pin fixation; T_4_, 15 min after skull pin fixation.

The primary outcomes of this study were the effective concentrations of dexmedetomidine associated with 50% and 95% probabilities of successful analgesia (EC_50_ and EC_95_), which were derived from the modified Dixon’s up-and-down sequential allocation method and probit regression analysis. For these calculations, each patient’s analgesic response to skull pin fixation was recorded as a binary outcome (success or failure) based on predefined criteria. The binary responses served as the input data for constructing the concentration–response curve and estimating EC_50_ and EC_95_. Secondary outcomes included serial MAP, HR, BIS, ANIi, and ANIm values at predefined time points.

### Statistical methods

Because this study employed the modified Dixon’s up-and-down sequential allocation method, conventional sample size or power analyses were not applicable. In this design, statistical adequacy is determined by the number of success–failure crossover reversals rather than by predefined power calculations. According to the methodological recommendation^24^, at least six independent reversals (success–failure pairs) are required to achieve a stable estimate of the median effective concentration (EC_50_); therefore, the sample size was determined based on this criterion. Recruitment continued until at least six independent reversal pairs were obtained, after which enrollment was terminated. Demographic data were presented as mean ± standard deviation or as number with percentage. Upon completion of the sequential allocation, the data were also analyzed using probit regression to estimate the effective concentrations associated with 50% (EC_50_) and 95% (EC_95_) probabilities of successful analgesia. The predicted dose–response curve was plotted, and the EC_50_ and EC_95_ values were derived along with their corresponding confidence intervals. All statistical analyses were performed using SPSS Statistics, version 27.0 (IBM Corp., Armonk, NY, USA).

## Results

**Fig. 1:**
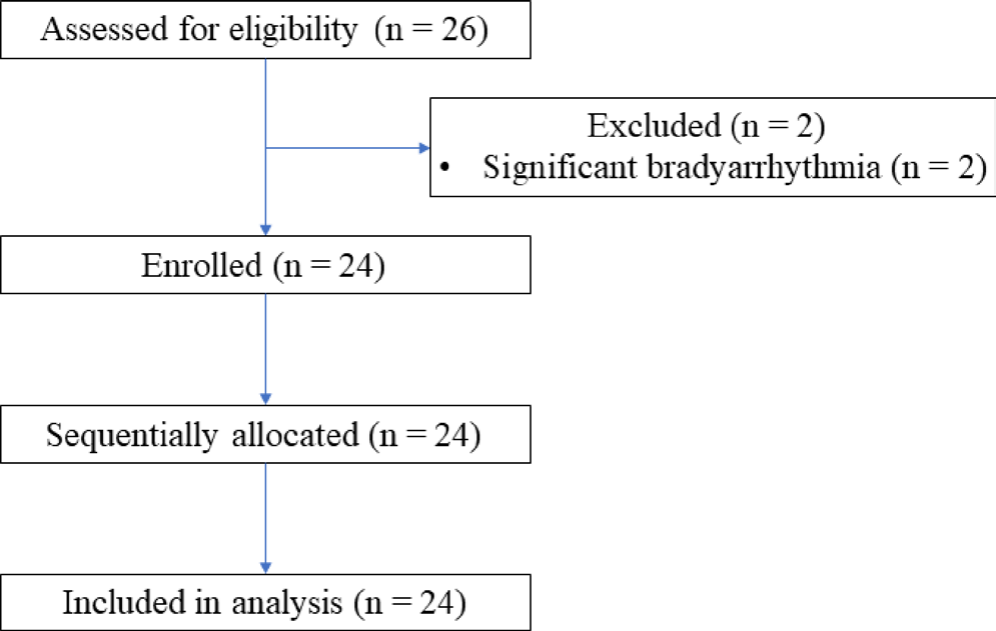
Flow diagram of patient recruitment according to the study protocol. Of the 26 patients assessed for eligibility, 2 were excluded due to significant bradyarrhythmia, resulting in 24 participants enrolled and included in the sequential allocation and analysis.

A total of 26 patients scheduled for elective intracranial surgery with skull pin fixation were assessed for eligibility, of whom 2 were excluded due to significant bradyarrhythmia. The remaining 24 patients were enrolled and sequentially allocated according to the modified Dixon’s up-and-down method. All enrolled participants completed the study and were included in the final analysis. The patient flow diagram is illustrated in Fig. 1. In the present study, the sequential allocation yielded more than six independent reversal pairs, meeting the predefined criterion for adequate estimation of EC_50_. At this point, the dose–response sequence demonstrated stable oscillation around the target concentration, indicating that the sample size was sufficient for a reliable EC_50_ estimate, and patient enrollment was therefore discontinued.

Patient demographics and clinical characteristics are summarized in Table 2. The mean age was 62.8 ± 9.2 years, with a male-to-female ratio of 16:8. Tumor locations were distributed as follows: pituitary (*n* = 7), frontal lobe (*n* = 6), occipital lobe (*n* = 3), cerebellum (*n* = 3), temporal lobe (*n* = 2), skull base (*n* = 2), and parietal lobe (*n* = 1). The mean durations of anesthesia and surgery were 262.4 ± 142.3 min and 200.7 ± 129.2 min, respectively. All patients had a preoperative Glasgow Coma Scale of 15.

**Table 2:** Overall patient demographics and clinical characteristics.

Variable	Value
Age (years), mean (SD)	62.8 (9.2)
Sex (male), n (%)	16 (66.7)
Height (cm), mean (SD)	160.4 (7.6)
Weight (kg), mean (SD)	64.0 (12.3)
Tumor location, n (%)	
Pituitary	7 (29.2)
Frontal lobe	6 (25.0)
Occipital lobe	3 (12.5)
Cerebellum	3 (12.5)
Temporal lobe	2 (8.3)
Skull base	2 (8.3)
Parietal lobe	1 (4.2)
Anesthesia time (min), mean (SD)	262.4 (142.3)
Operation time (min), mean (SD)	200.7 (129.2)

Data are expressed as mean ± standard deviation or number with percentage. SD, standard deviation.

Serial measurements around skull pin fixation revealed significant changes in both hemodynamic and neurophysiological parameters (Table 3). MAP showed significant reductions at T_1_, T_3_, and T_4_ compared to baseline and intra-fixation values (*p* < 0.001). HR decreased significantly following fixation, from 72.3 ± 11.1 bpm at baseline (T_0_) to 62.3 ± 10.2 bpm at 15 min post-fixation (T_4_; *p* = 0.037). ANI values also declined markedly during skull pin fixation. The ANIi dropped from 76.8 ± 13.4 at T_0_ to 42.3 ± 19.9 at T_2_ (*p* < 0.001), while the ANIm decreased from 78.3 ± 10.5 to 55.4 ± 17.5 over the same period (*p* = 0.034). Furthermore, BIS values were significantly lower at all post-induction time points (T_1_–T_4_) compared to T_0_ (*p* < 0.001). Correspondingly, the Ce of propofol and remifentanil, as well as the Cp of dexmedetomidine, exhibited significant changes throughout the time points (*p* < 0.001).

**Table 3:** Serial measurements of hemodynamic and neurophysiological parameters and drug concentrations at predefined time points around skull pin fixation.

Variable	T_0_	T_1_	T_2_	T_3_	T_4_	*p* value
MAP (mmHg)	105.2 ± 10.4	90.0 ± 14.0#*	107.7 ± 19.1	88.4 ± 15.5#*	87.4 ± 16.0#*	< 0.001
HR (bpm)	72.3 ± 11.1	66.5 ± 12.6	68.1 ± 12.2	63.0 ± 10.5	62.3 ± 10.2#	0.037
ANIi	76.8 ± 13.4*	69.3 ± 16.3*	42.3 ± 19.9#	67.9 ± 15.5*	69.5 ± 18.9*	< 0.001
ANIm	78.3 ± 10.5*	66.8 ± 17.0*	55.4 ± 17.5#	68.3 ± 13.4*	67.4 ± 16.4*	0.034
BIS	93.1 ± 4.1*	45.3 ± 7.2#	45.9 ± 5.2#	44.9 ± 5.8#	44.6 ± 7.6#	< 0.001
Ce of propofol (mcg/mL)	3.8 ± 0.9*	2.2 ± 0.4#	2.1 ± 0.4#	2.0 ± 0.4#	2.0 ± 0.4#	< 0.001
Ce of remifentanil (ng/mL)	2.3 ± 0.7*	1.3 ± 0.8#*	2.0 ± 0.0#	1.0 ± 0.5#*	1.0 ± 0.6#*	< 0.001
Cp of dexmedetomidine (ng/mL)	0.00 ± 0.00*	0.20 ± 0.02#*	0.33 ± 0.04#	0.20 ± 0.02#*	0.20 ± 0.00#*	< 0.001

Data are expressed as mean ± standard deviation. ANIi, instantaneous analgesia nociception index; ANIm, mean analgesia nociception index; BIS, bispectral index; bpm, beats per minute; Ce, effect-site concentration; Cp, plasma concentration; HR, heart rate, MAP, mean arterial pressure. T_0_: baseline; T_1_: 2 min before pin fixation; T_2_: during pin fixation; T_3_: 5 min after pin fixation; T_4_: 15 min after pin fixation. #, *p* < 0.05 between T_0_ and other time points; *, *p* < 0.05 between T_2_ and other time points.

When patients were divided into two groups based on the presence or absence of successful analgesia during skull pin fixation (*n* = 13 vs. *n* = 11), significant differences were observed (Table 4). Compared to the failure group, patients in the successful analgesia group exhibited significantly lower MAP (99.8 ± 20.2 vs. 117.0 ± 13.2 mmHg, *p* = 0.02), higher ANIi values (50.4 ± 15.3 vs. 32.3 ± 21.1, *p* = 0.03), and a smaller reduction in ANIi (ΔANIi; − 17.7 ± 17.6 vs. − 38.1 ± 20.5, *p* = 0.018). Similarly, the reduction in ANIm (ΔANIm) was significantly smaller in the successful group (− 2.4 ± 17.1 vs. − 22.0 ± 16.5, *p* = 0.009). No significant differences were observed in BIS values, changes in BIS values (ΔBIS), or the Ce of propofol between the two groups. However, the successful group received a significantly higher Cp of dexmedetomidine (0.36 ± 0.04 vs. 0.30 ± 0.04 ng/mL, *p* < 0.001).

**Table 4:** Comparison of hemodynamic and neurophysiological parameters and drug concentrations between successful and unsuccessful analgesia during skull pin fixation.

Variable	Successful (*n* = 13)	Unsuccessful (*n* = 11)	*p* value
MAP (mmHg)	99.8 ± 20.2*	117.0 ± 13.2	0.020
ΔMAP (mmHg)	13.6 ± 12.4	22.5 ± 13.9	0.115
HR (bpm)	64.8 ± 12.4	72.0 ± 11.1	0.150
ΔHR (bpm)	− 0.1 ± 7.9	3.4 ± 4.9	0.197
BIS	46.7 ± 4.9	45.1 ± 5.7	0.480
ΔBIS	1.6 ± 6.4	− 0.1 ± 8.9	0.598
ANIi	50.4 ± 15.3*	32.3 ± 21.1	0.030
ΔANIi	− 17.7 ± 17.6*	− 38.1 ± 20.5	0.018
ANIm	61.4 ± 12.2	48.3 ± 20.6	0.070
ΔANIm	− 2.4 ± 17.1**	− 22.0 ± 16.5	0.009
Ce of propofol (mcg/mL)	2.2 ± 0.4	2.1 ± 0.3	0.360
ΔCe of propofol (mcg/mL)	0.1 ± 0.15	0.1 ± 0.19	0.760
Cp of dexmedetomidine (ng/mL)	0.36 ± 0.04**	0.30 ± 0.04	0.001

Data are expressed as mean ± standard deviation. ANIi, instantaneous analgesia nociception index; ANIm, mean analgesia nociception index; BIS, bispectral index; bpm, beats per minute; Ce, effect-site concentration; Cp, plasma concentration; HR, heart rate, MAP, mean arterial pressure. Δ, change from the time point immediately before to during skull pin fixation. *, *p* < 0.05 between the successful and failed analgesia groups; **, *p* < 0.01 between the successful and failed analgesia groups.

**Fig. 2:**
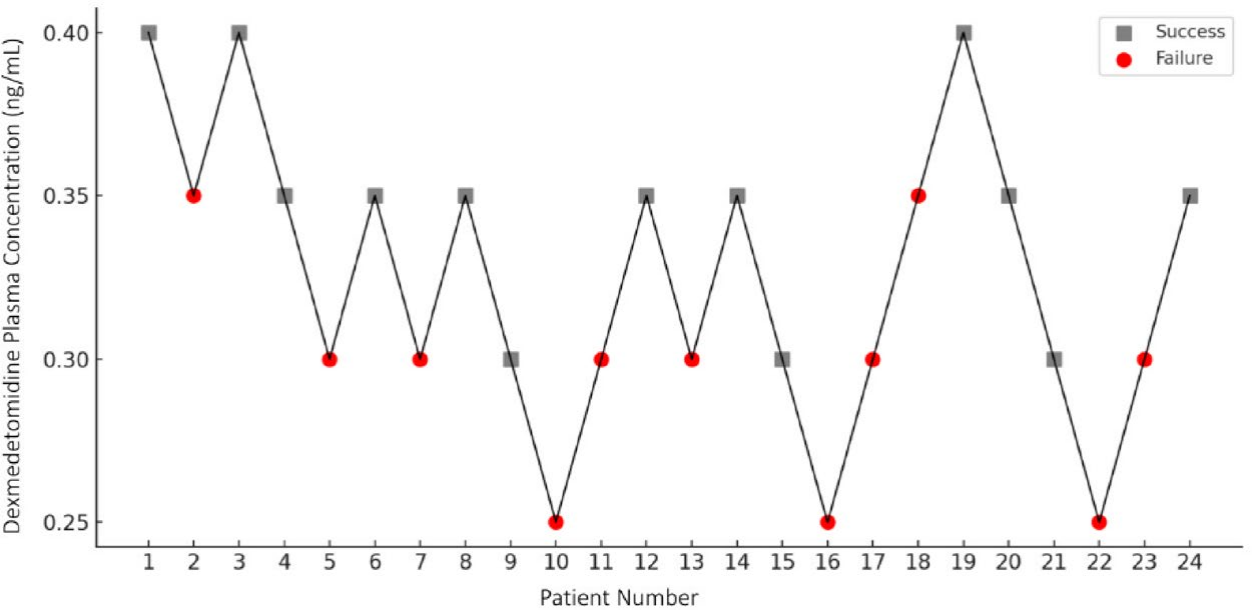
Up-and-down sequential allocation of dexmedetomidine plasma concentrations. Each dot represents an individual patient’s outcome, with squares (■) indicating successful analgesia and circles (●) indicating analgesic failure. The blue line connects patients in sequence according to the modified Dixon’s up-and-down method. Oscillations around the effective concentration threshold are evident, supporting the identification of the concentration–response transition zone.

**Fig. 3:**
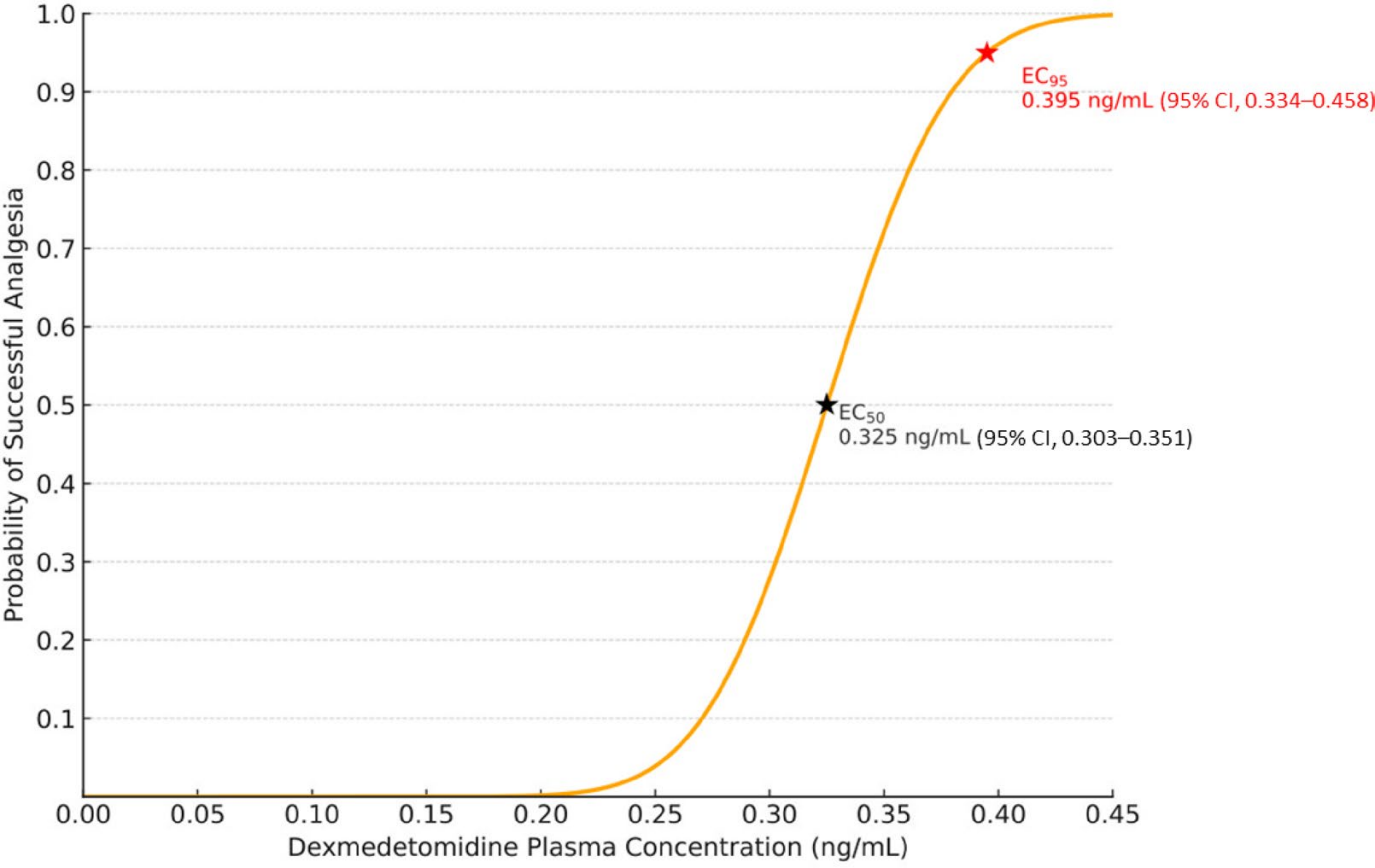
Probit regression dose–response curve for dexmedetomidine. The fitted curve illustrates the predicted probability of successful analgesia as a function of dexmedetomidine plasma concentrations. The EC_50_ (0.325 ng/mL) and EC_95_ (0.395 ng/mL) are marked with asterisks and labeled accordingly next to the curve. CI, confidence interval.

The distribution of dexmedetomidine Cp associated with successful or failed skull pin fixation for each consecutive patient is presented in Fig. 2. The sequential dataset used for probit regression, including all dexmedetomidine concentrations, analgesic responses (success/failure), and the reversal pairs used to estimate EC_50_, has been included in Supplementary Table S1. Based on probit regression analysis, the estimated EC_50_ and EC_95_ values for achieving successful analgesia were 0.325 ng/mL (95% confidence interval, 0.303–0.351) and 0.395 ng/mL (95% confidence interval, 0.334–0.458), respectively, as shown in Fig. 3. The fitted dose–response curve demonstrated a typical sigmoidal shape, reflecting a smooth and progressive increase in the probability of success with increasing dexmedetomidine concentrations.

## Discussion

In this study, we determined the effective Cp of dexmedetomidine required to achieve successful analgesia during skull pin fixation using a modified up-and-down sequential allocation design followed by probit regression analysis. The estimated EC_50_ and EC_95_ values were 0.325 ng/mL and 0.395 ng/mL, respectively. These results provide an evidence-based concentration range to guide the clinical titration of dexmedetomidine, optimizing analgesic efficacy while minimizing the risk of adverse effects.

Skull pin fixation during intracranial surgery elicits a marked sympathetic response due to intense periosteal nociceptive stimulation. Several studies have investigated the efficacy of intravenous dexmedetomidine at varying doses in attenuating the hemodynamic response to skull pin application. El Dawlatly et al. reported that a low dose of dexmedetomidine (0.25 mcg/kg over 10 min; estimated peak Cp: ~ 0.15 ng/mL) was as effective as lidocaine infiltration in blunting the hemodynamic response to skull pin fixation, with minimal adverse effects^22^. On the other hand, a higher dose (1 mcg/kg over 10 min; estimated peak Cp: ~ 0.6 ng/mL) has demonstrated greater efficacy in suppressing the stress response, but is more frequently associated with hypotension and bradycardia^10–12^. A previous meta-analysis further supported that dexmedetomidine was more effective than fentanyl in blunting skull pin-induced increases in MAP and HR^25^. Notably, despite the administration of dexmedetomidine, additional interventions such as opioids, esmolol, or increased concentrations of volatile anesthetics were still required in 23.3–33.3% of patients to control hypertension or tachycardia^12^. These findings suggest that the efficacy of dexmedetomidine may be dose-dependent and frequently inadequate as monotherapy. Moreover, prior studies typically involved the use of inhalational anesthetics, often combined with nitrous oxide^10–12,22^, which likely provided additional analgesic effects and potentially confounded the true contribution of dexmedetomidine to nociceptive control. In contrast, our study excluded these volatile agents by using TIVA with propofol and a fixed remifentanil Ce of 2.0 ng/mL, allowing a more accurate assessment of the analgesic and sympatholytic properties of dexmedetomidine. This approach also minimized potential adverse effects and reduced the likelihood of under-treatment, thereby enhancing overall patient safety during skull pin fixation.

TCI is an advanced system for delivering intravenous agents such as propofol and remifentanil, using pharmacokinetic models to maintain target Cp or Ce^26^. By real-time adjustment of infusion rates, TCI enhances the precision, consistency, and control of anesthesia, making it valuable for both clinical use and pharmacological research^27^. The pharmacokinetics of dexmedetomidine can be described by a three-compartment model, with the Dyck model—a preliminary framework using height as the sole covariate—allowing a target Cp to be set via a TCI pump^28^. While sedation and analgesia should ideally be titrated to a target Ce rather than a target Cp, effect-site TCI models for dexmedetomidine have not yet been widely adopted in clinical practice; therefore, plasma-site TCI remains the most practical alternative. Although no prior study has reported the effects of dexmedetomidine administered via TCI in patients undergoing skull pin fixation compared to manual infusion techniques, the use of TCI may offer potential benefits. Due to the relatively slow pharmacokinetic profile of dexmedetomidine, fixed-rate manual infusions can lead to a gradual rise in Cp over time. In contrast, TCI guided by a validated pharmacokinetic model allows for more precise control of drug delivery, helping to maintain stable and predictable Cp^29^, particularly in situations where the timing of skull pin application is uncertain. Additionally, TCI of dexmedetomidine eliminates the need to manually calculate a loading dose, as the model automatically adjusts for this when targeting the desired Cp, thereby reducing the risk of dosing errors. In our study, dexmedetomidine was administered using a TCI system to achieve and maintain a target Cp, resulting in consistent analgesic and hemodynamic effects during skull pin fixation.

Among the 11 patients in the unsuccessful analgesia group, 8 exhibited an ANIi value < 30 during skull pin fixation, indicating a pronounced nociceptive response. Two patients developed isolated hypertension without concurrent tachycardia, and were therefore not classified as having a hyperdynamic response. Notably, one patient presented with both hypertension and an ANIi value < 30, reflecting a convergence of hemodynamic instability and reduced nociceptive tolerance. The sensitivity and specificity of an ANIi value < 30 for detecting analgesia failure during skull pin fixation were 81.8% and 100%, respectively. This finding indicates that an ANI threshold of 30 serves as a highly specific marker of inadequate analgesia, with only two false-negative cases observed. No patients in the successful analgesia group exhibited an ANIi < 30, underscoring the potential of ANI monitoring as a reliable adjunct for nociceptive assessment during neurosurgical procedures.

Previous studies have demonstrated a negative correlation between the ANI and hemodynamic parameters such as MAP and HR during skull pin fixation^18,19^. In our study, we further confirmed that elevations in MAP and HR were associated with decreases in ANI, reinforcing the inverse relationship between autonomic nociceptive responses and ANI values. ANI < 30 and MAP/HR variations exceeding 20% from baseline were used as criteria for inadequate analgesia in this study. Our results indicate that dexmedetomidine at a Cp of 0.395 ng/mL, combined with remifentanil at a Ce of 2.0 ng/mL, effectively maintained ANI ≥ 30 and hemodynamic stability in 95% of patients during skull pin fixation. However, patients who received dexmedetomidine Cp below 0.3 ng/mL experienced significantly greater fluctuations in MAP and HR, suggesting that ANI alone may not fully reflect the hemodynamic consequences of noxious stimulation. Dexmedetomidine reduces HR by enhancing vagal activity and increasing parasympathetic tone^30^, which contributes to elevated baseline ANI values. Following a noxious stimulus, the diminished respiratory influence on heart rate variability (HRV), combined with the initially elevated ANI, may ultimately delay the detection of a full nociceptive response^31^. As a result, the impact of dexmedetomidine on HRV can compromise the precision of ANI in reflecting nociceptive responses. Notably, a recent randomized controlled study reported that ANI may provide greater sensitivity to nociceptive changes during dexmedetomidine-based anesthesia, although with a delayed response during skull pin fixation^20^. These findings underscore the value of integrating ANI with conventional hemodynamic monitoring. While ANI provides valuable insights into nociceptive states, it should not be used in isolation. A multimodal monitoring strategy offers a more comprehensive and reliable assessment of anesthetic depth and analgesic adequacy.

Concerns related to population heterogeneity, particularly variations in tumor location, and the potential influence of laryngeal stimulation may have introduced bias into the primary outcomes in our study. Notably, although patients presented with intracranial tumors in various regions, skull pin fixation generates a uniform periosteal nociceptive stimulus that is physiologically independent of tumor distribution; thus, the impact of tumor-related heterogeneity on the nociceptive response to pinning is likely minimal. In addition, the potential confounding effect of endotracheal tube-related laryngeal stimulation was minimized through strict standardization. All patients were intubated under deep anesthesia with full neuromuscular blockade, and head positioning was completed before the target dexmedetomidine concentration was reached. No further manipulation of the endotracheal tube occurred during skull pin fixation, ensuring that the sympathetic responses observed at the time of pinning predominantly reflected the nociceptive stimulus itself rather than laryngeal stimulation. Overall, these potential sources of bias cannot be completely excluded, but the standardized anesthetic and surgical procedures used in this study may have reduced their impact to the minimum extent possible.

Several limitations should be acknowledged. First, although the up-and-down method was efficient for estimating the EC_50_, its relatively small sample size limited the precision of higher percentile estimates, such as the EC_95_. Nonetheless, it effectively identified the median effective concentration while minimizing patient exposure to potentially inadequate dosing. The consistent oscillations observed in the sequential dose allocation supported the method’s ability to capture the concentration–response transition zone. Additionally, the probit regression produced a smooth, sigmoid dose–response curve, consistent with the typical pharmacodynamic profile of dexmedetomidine. Second, the influence of concomitant remifentanil infusion on dexmedetomidine’s analgesic requirements must be considered when interpreting these results. Therefore, the findings may not be directly generalizable to clinical scenarios in which dexmedetomidine is used as a sole agent. Further investigation incorporating pharmacokinetic–pharmacodynamic modeling and varying opioid backgrounds could help refine dexmedetomidine dosing strategies. Third, a fixed threshold (ANI < 30) was used to define nociceptive failure during skull pin fixation in our study. Although this absolute cutoff has been widely used in previous studies^19,32^, a relative decrease in ANIi or ANIm of more than 10 points from baseline within 1 min of skull pin fixation may also indicate nociceptive activation or sympathetic responses^33^. By relying solely on a fixed threshold, subtle nociceptive responses that did not meet the criterion may have been overlooked. Future studies should consider incorporating both absolute and relative ANI changes to more sensitively detect nociceptive events during neurosurgical procedures.

## Conclusions

With remifentanil maintained at a fixed Ce of 2.0 ng/mL, the optimal Cp of dexmedetomidine for achieving both hemodynamic and nociceptive stability was identified as 0.395 ng/mL. These findings underscore the value of ANI monitoring and individualized dexmedetomidine titration in optimizing intraoperative anesthesia management.

## Supplementary Information

Below is the link to the electronic supplementary material.

Supplementary Material 1

## Data Availability

The data analyzed during the current study are available from the corresponding author on reasonable request.
