# Supplementary material for: Plasma concentration of dexmedetomidine combined with fixed remifentanil for nociceptive and hemodynamic control during skull pin fixation
Source: Sci Rep. 2026 Apr 27;16:19441. doi: 10.1038/s41598-026-50692-y (PMC13287627; doi:10.1038/s41598-026-50692-y)
Supplement: Supplementary file 1 — Supplementary Material 1 [file 41598_2026_50692_MOESM1_ESM.docx]

| **Supplementary Table S1.** Sequential Allocation Dataset and Reversal Pairs | | | |
| --- | --- | --- | --- |
| Patient Number | Dexmedetomidine Cp (ng/mL) | Response (Success/Failure) | Reversal (Yes/No) |
| 1 | 0.40 | Success | No |
| 2 | 0.35 | Failure | Yes (R1) |
| 3 | 0.40 | Success | Yes (R2) |
| 4 | 0.35 | Success | No |
| 5 | 0.30 | Failure | Yes (R3) |
| 6 | 0.35 | Success | Yes (R4) |
| 7 | 0.30 | Failure | Yes (R5) |
| 8 | 0.35 | Success | Yes (R6) |
| 9 | 0.30 | Success | No |
| 10 | 0.25 | Failure | Yes (R7) |
| 11 | 0.30 | Failure | No |
| 12 | 0.35 | Success | Yes (R8) |
| 13 | 0.30 | Failure | Yes (R9) |
| 14 | 0.35 | Success | Yes (R10) |
| 15 | 0.30 | Success | No |
| 16 | 0.25 | Failure | Yes (R11) |
| 17 | 0.30 | Failure | No |
| 18 | 0.35 | Failure | No |
| 19 | 0.40 | Success | Yes (R12) |
| 20 | 0.35 | Success | No |
| 21 | 0.30 | Success | No |
| 22 | 0.25 | Failure | Yes (R13) |
| 23 | 0.30 | Failure | No |
| 24 | 0.35 | Success | Yes (R14) |

Cp, plasma concentration; R, reversal.
